# Supplementary material for: Evidence supported by Mendelian randomization: impact on inflammatory factors in knee osteoarthritis
Source: Front Med (Lausanne). 2024 May 28;11:1382836. doi: 10.3389/fmed.2024.1382836 (PMC11165061; doi:10.3389/fmed.2024.1382836)
Supplement: Supplementary file 1 [file Data_Sheet_1.ZIP › data/GCST90274786_5e6/786.leaveoneout.pdf]

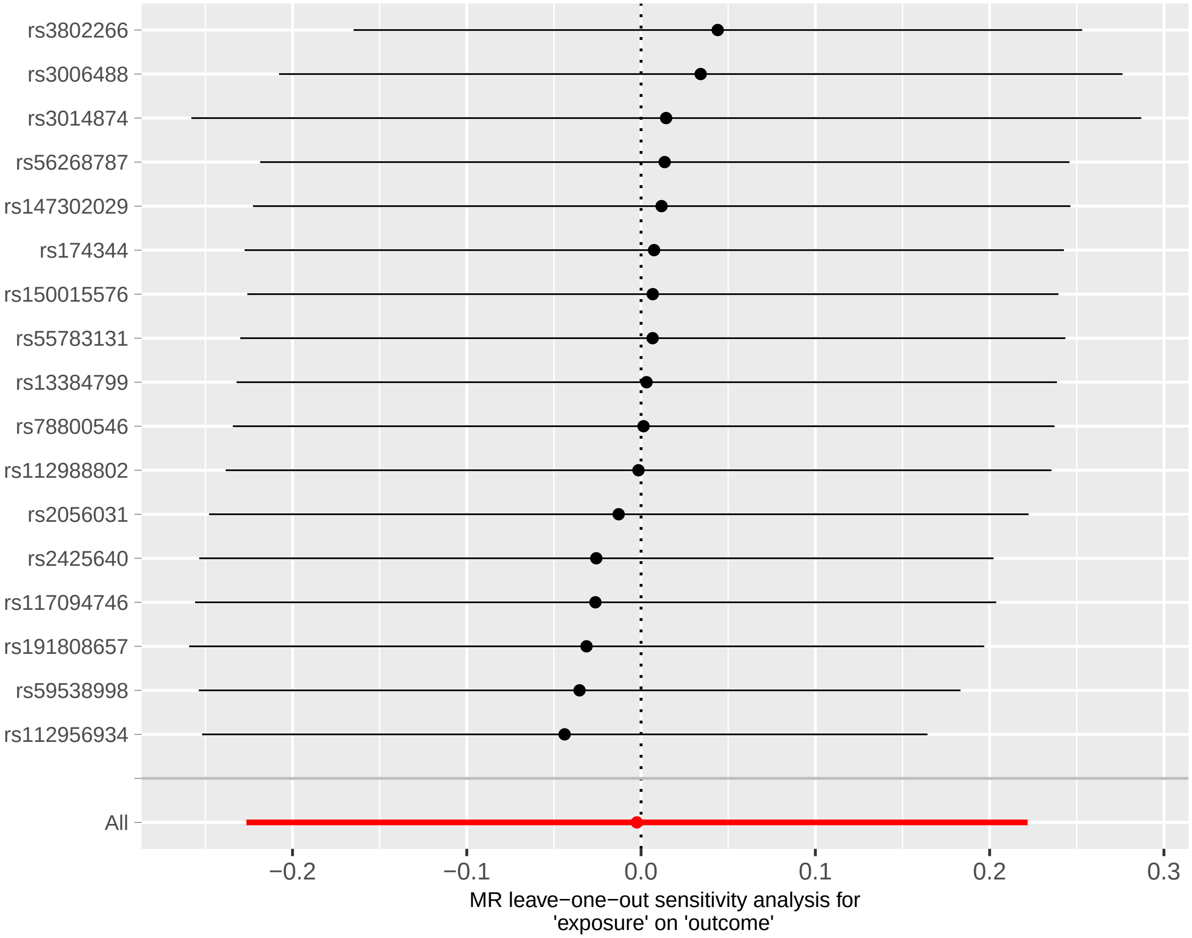
rs3802266
rs3006488
rs3014874
rs56268787
rs147302029
rs174344
rs150015576
rs55783131
rs13384799
rs78800546
rs112988802
rs2056031
rs2425640
rs117094746
rs191808657
rs59538998
rs112956934
All
−0.2
−0.1
0.0
0.1
0.2
0.3
MR leave−one−out sensitivity analysis for
'exposure' on 'outcome'
